# Supplementary material for: Human Brain Ancestral Barcodes
Source: bioRxiv. 2024 Nov 12:2024.07.14.603450. Originally published 2024 Jul 17. Preprint. [Version 2] doi: 10.1101/2024.07.14.603450 (PMC11275915; doi:10.1101/2024.07.14.603450)
Supplement: 1 [file NIHPP2024.07.14.603450V2-supplement-1.pdf]

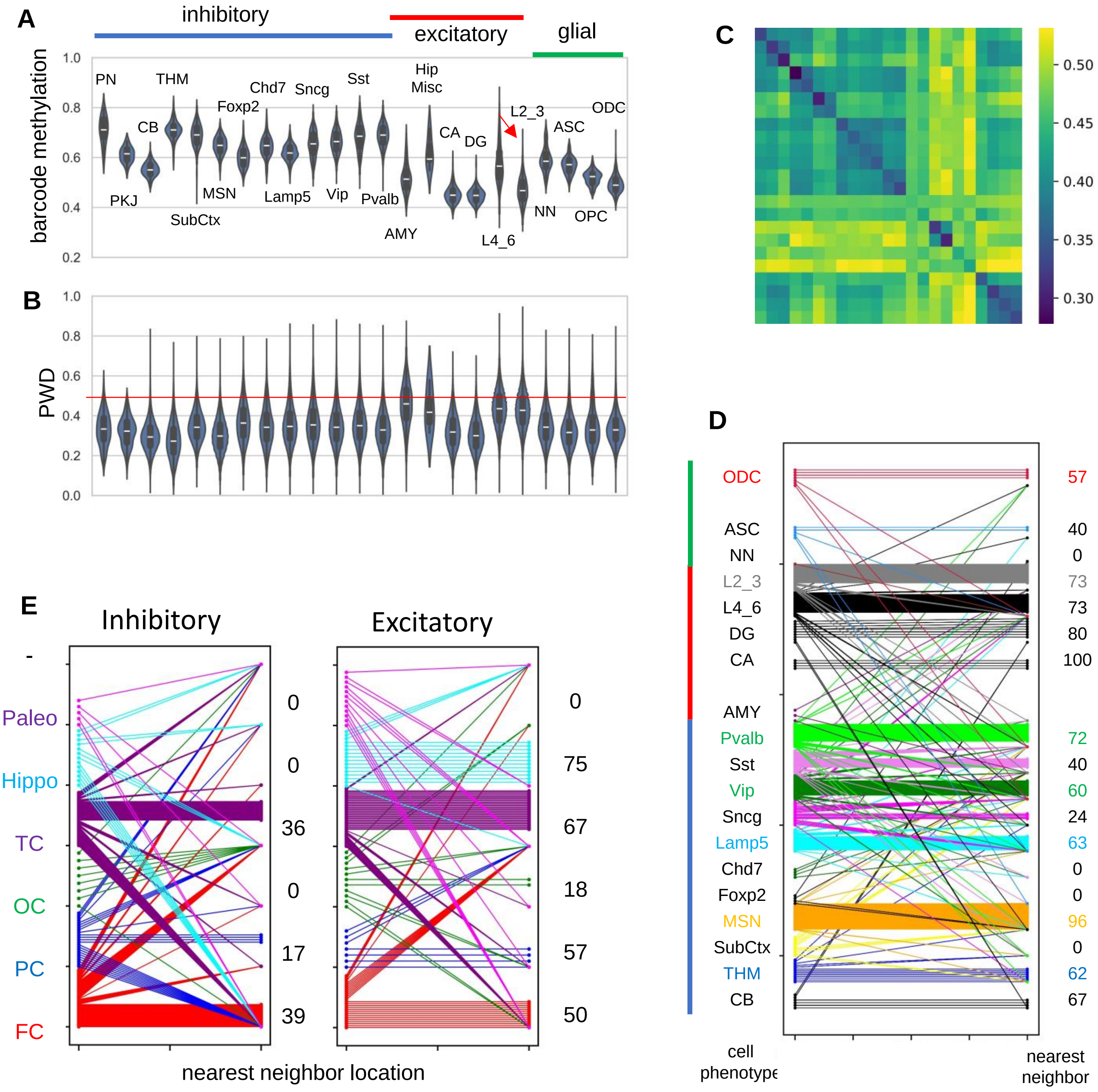

Supplemental Figure 1: H02 data

A) Barcode methylation for different cell types

B) PWDs between cells of the same type

C) PWDs between cell types

D) Lineage cell type fidelity between nearest neighbor pairs (PWD<0.05)

E) Location fidelity between nearest neighbor pairs (PWD<0.05)

F

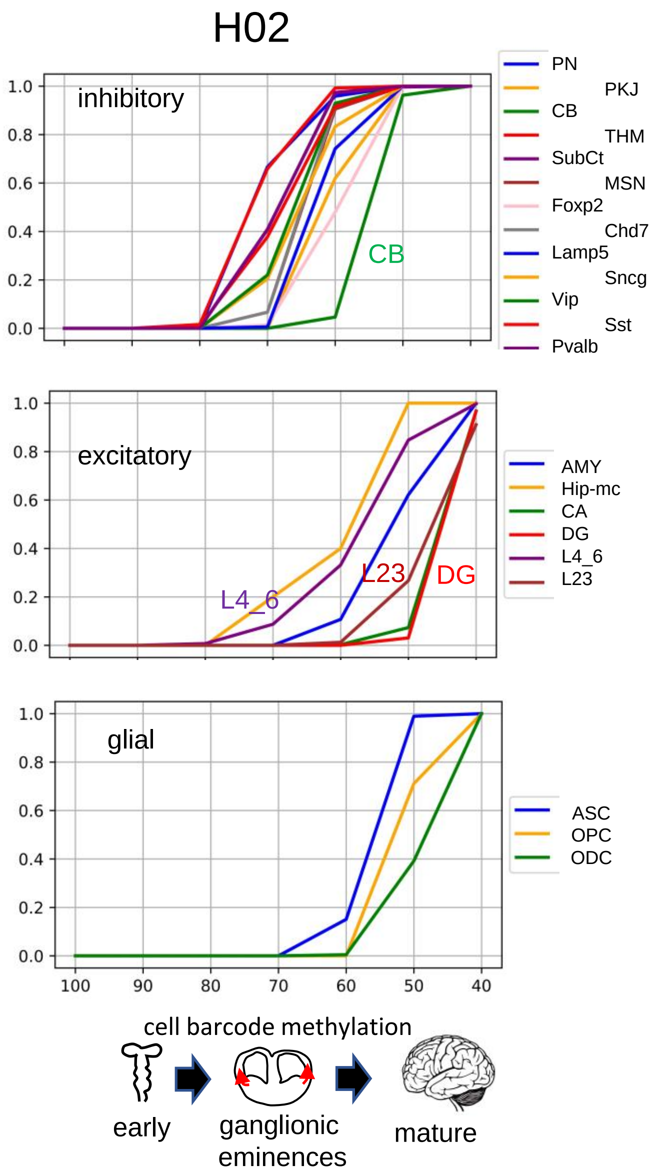

Supplemental Figure 1: H02 data

F) Barcode methylation versus final adult brain content indicates that inhibitory neurons appear first and reach their adult levels before excitatory or glial cells.

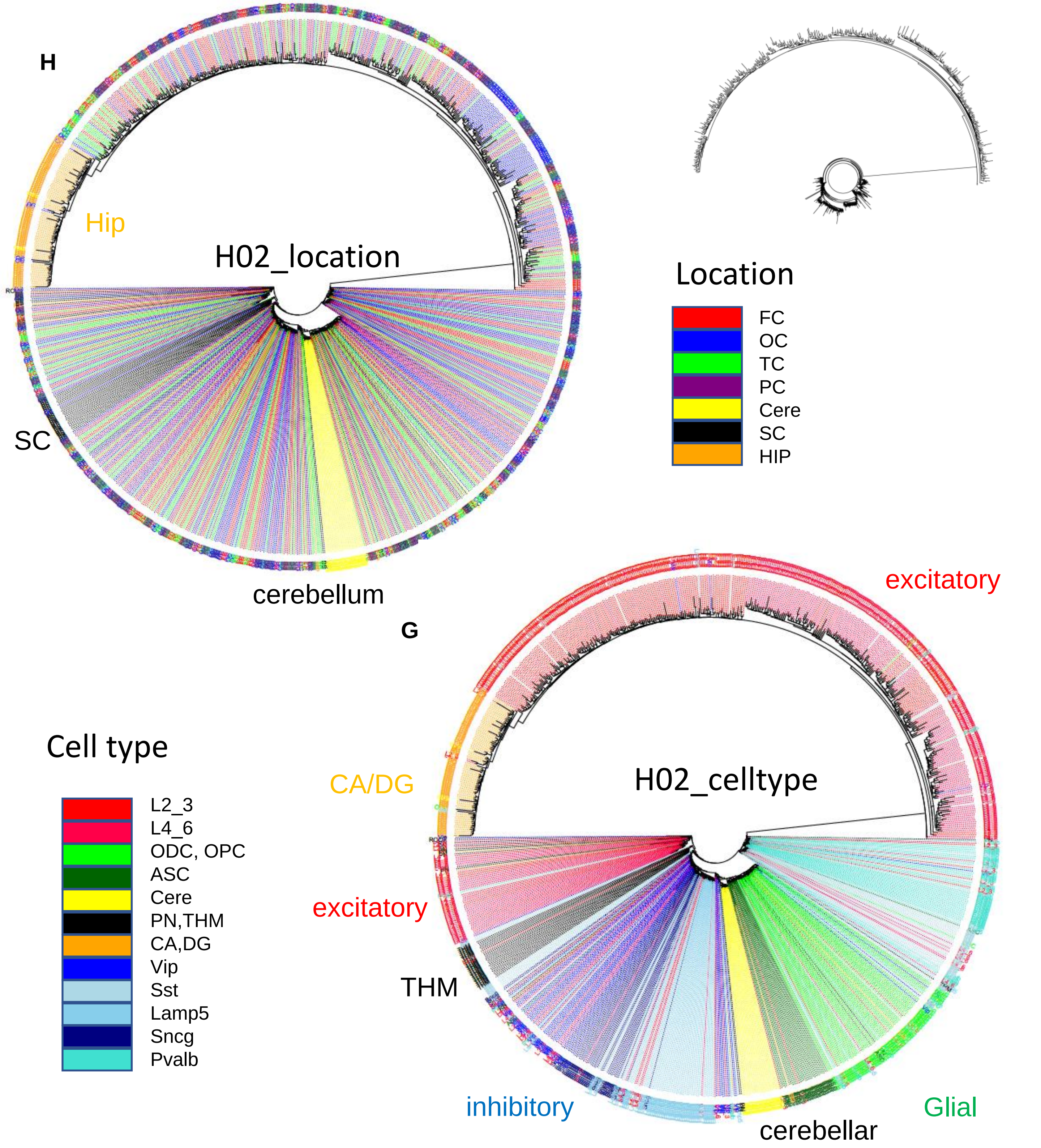

Supplemental Figure 1: H02 data

G) Ancestral tree with 1,001 cells rooted at a fully methylated progenitor shows sequential branching with excitatory, then brain stem, inhibitory and cerebellar neurons, then glial cells, and finally excitatory neurons with hippocampal neurons at the end.

H) Related cells colocalize for brain stem, cerebellar, and hippocampal neurons. Inhibitory neurons are more scattered. Excitatory neurons are also scattered with some localization within the cortex (see I & J for trees with more neurons)

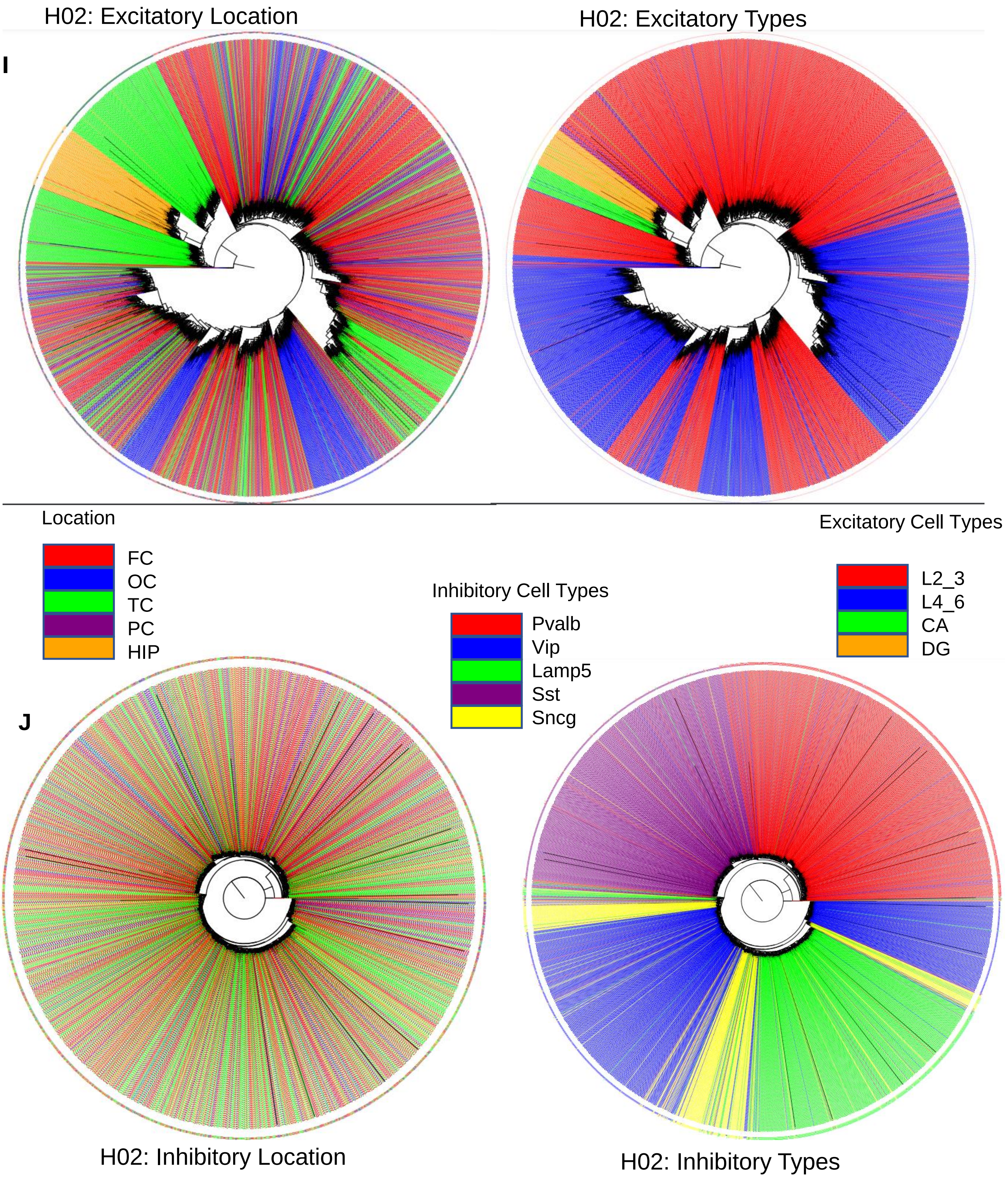

Supplemental Figure 1: H02 data

I) Ancestral tree with more (2,806) excitatory neurons shows more localization between related neurons in the hippocampus, occipital and temporal cortex. Related neurons also tend to have similar phenotypes.

J) Ancestral tree with more (2,788) inhibitory neurons still shows scattering between related neurons. Related neurons tend to have similar phenotypes.

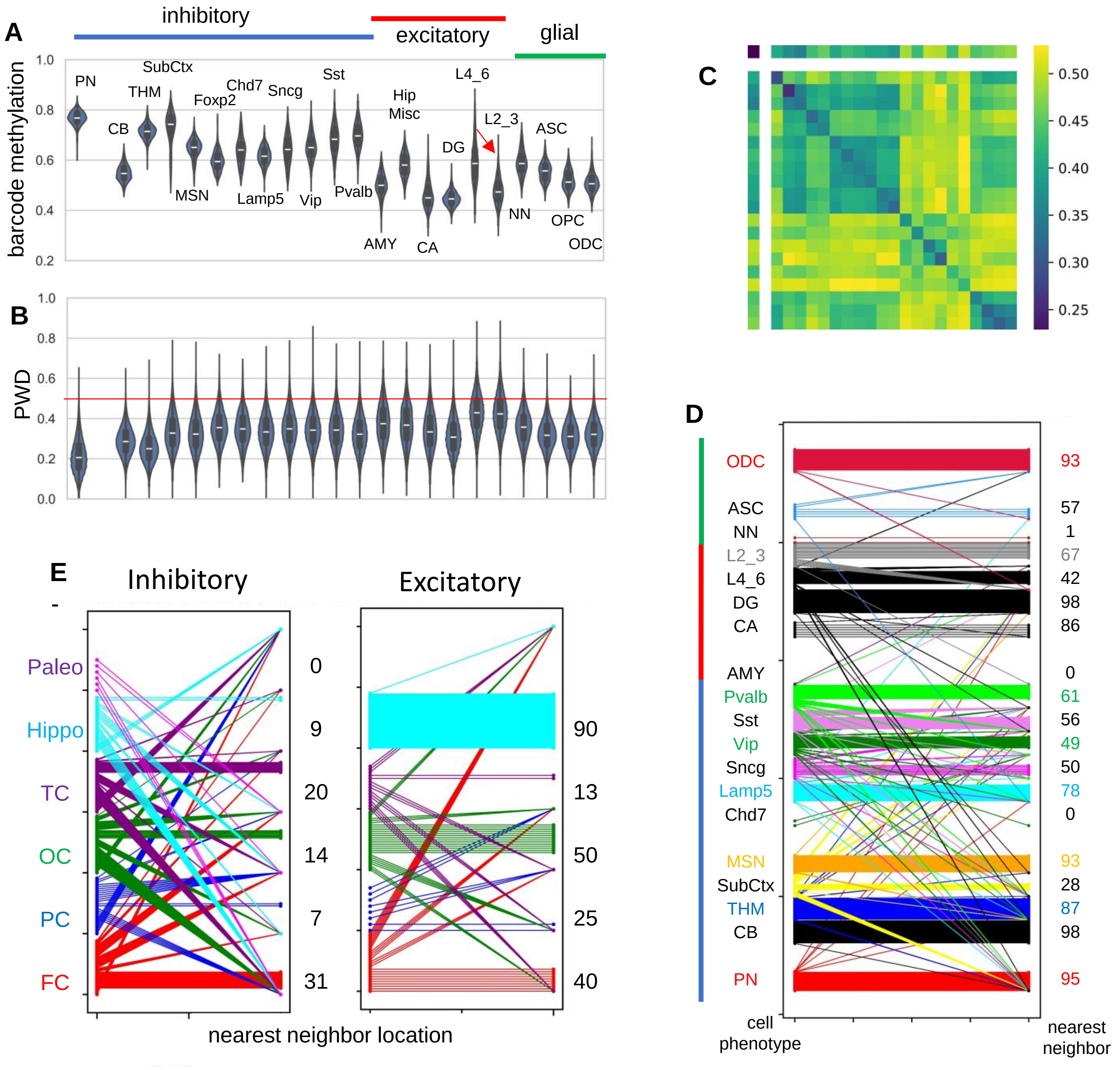

Supplemental Figure 2: H04 data

A) Barcode methylation for different cell types

B) PWDs between cells of the same type

C) PWDs between cell types

D) Lineage cell type fidelity between nearest neighbor pairs (PWD<0.05)

E) Location fidelity between nearest neighbor pairs (PWD<0.05)

F

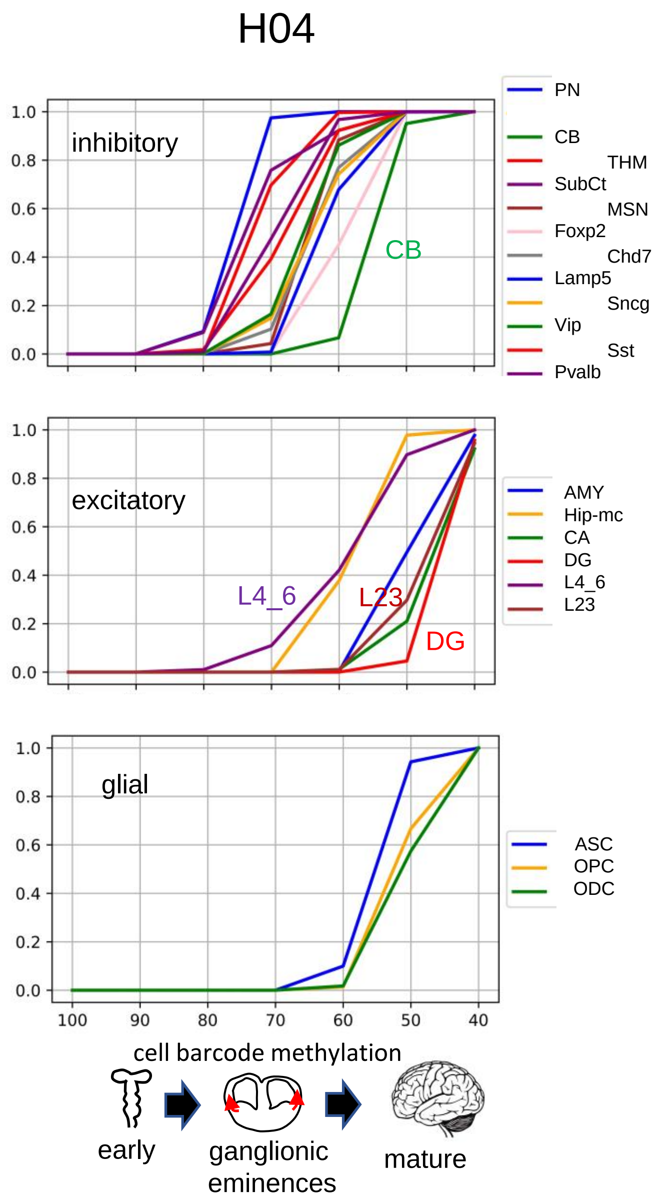

Supplemental Figure 2: H04 data

F) Barcode methylation versus final adult brain content indicates that inhibitory neurons appear first and reach their adult levels before excitatory or glial cells.

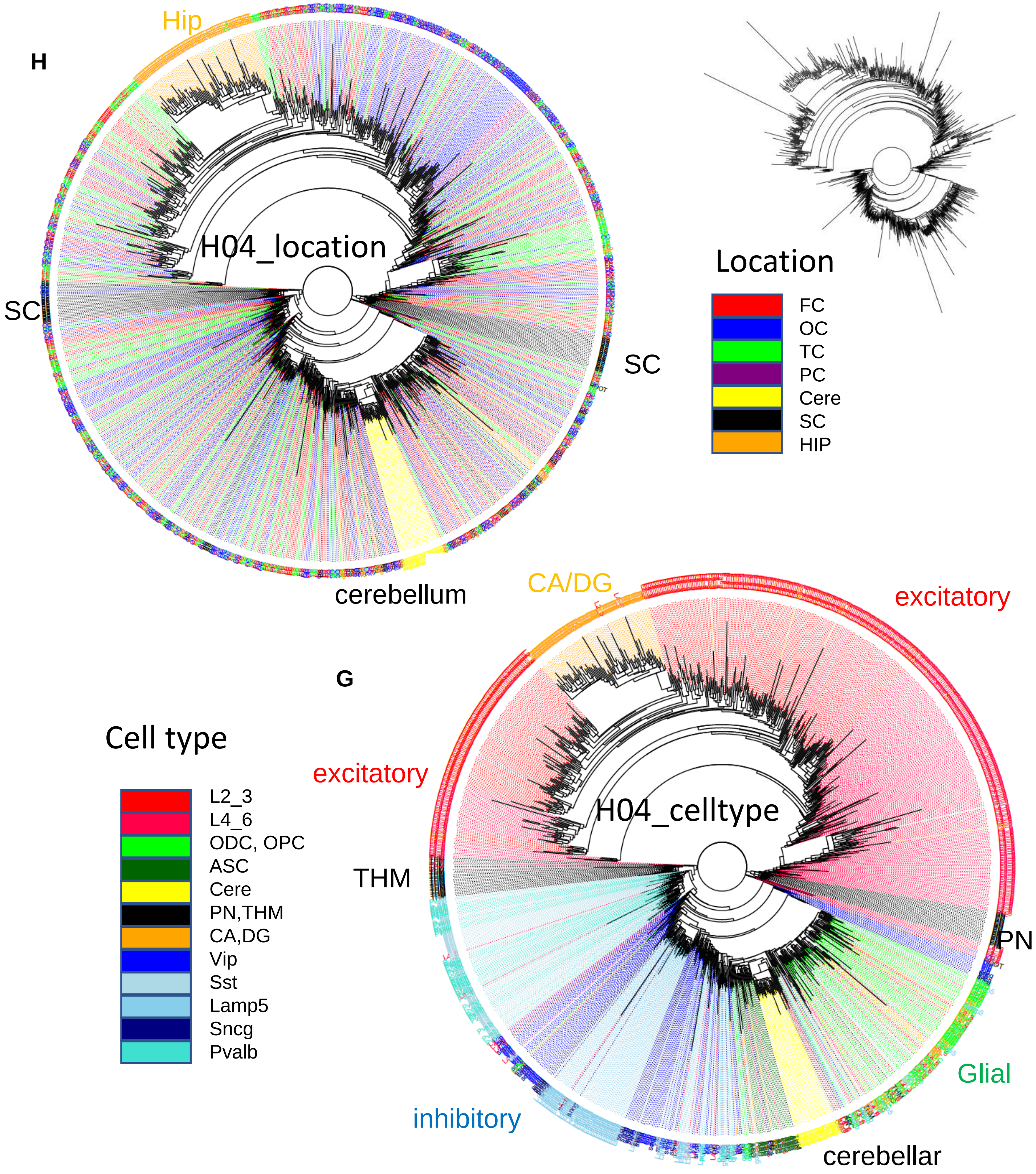

Supplemental Figure 2: H04 data

G) Ancestral tree with 1,033 cells rooted at a fully methylated progenitor shows sequential branching with excitatory, then brain stem, inhibitory and cerebellar neurons, then glial cells, and finally excitatory neurons with hippocampal neurons at the end.

H) Related cells colocalize for brain stem, cerebellar, and hippocampal neurons. Inhibitory neurons are more scattered. Excitatory neurons are also scattered with some localization within the cortex (see I & J for trees with more neurons)

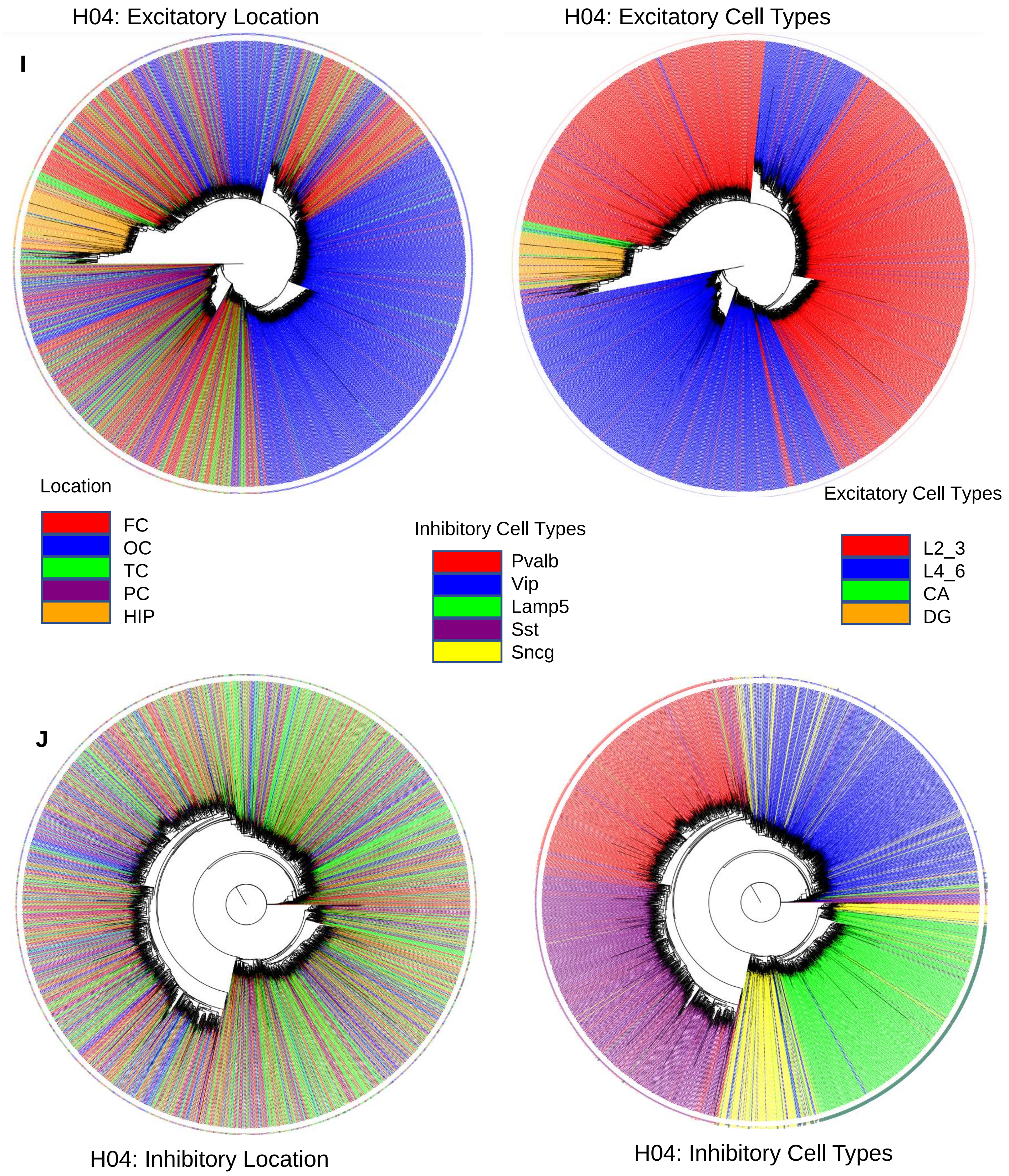

Supplemental Figure 2: H04 data

I) Ancestral tree with more (2,797) excitatory neurons shows more localization between related neurons in the hippocampus, occipital and temporal cortex. Related neurons also tend to have similar phenotypes.

J) Ancestral tree with more (2,752) inhibitory neurons still shows scattering between related neurons. Related neurons tend to have similar phenotypes.
