# Supplementary material for: Human Brain Barcodes
Source: bioRxiv. 2024 Jul 17:2024.07.14.603450. Preprint. [Version 1] doi: 10.1101/2024.07.14.603450 (PMC11275915; doi:10.1101/2024.07.14.603450)
Supplement: Supplementary file 1 [file NIHPP2024.07.14.603450V1-supplement-1.pdf]

bioRxiv preprint doi: https://doi.org/10.1101/2024.07.14.603450; this version posted July 17, 2024. The copyright holder for this preprint (which was not certified by peer review) is the author/funder, who has granted bioRxiv a license to display the preprint in perpetuity. It is made available under aCC-BY 4.0 International license.

Supplemental File 1: fCpG annotations from QDNAseq
Supplemental File 2: list of fCpG sites and basic data summaries for H01, H02 and H04
Supplemental File 3: H01 cells and methylation
Supplemental File 4: H02 cells and methylation
Supplemental File 5: H04 cells and methylation
Supplemental File 6: Cells used with IQtree

Supplemental Figure 1: H02 figures
Supplemental Figure 2: H04 figures
Supplemental Figure 3: Brain tumor data

bioRxiv preprint doi: https://doi.org/10.1101/2024.07.14.603450; this version posted July 17, 2024. The copyright holder for this preprint (which was not certified by peer review) is the author/funder, who has granted bioRxiv a license to display the preprint in perpetuity. It is made available under aCC-BY 4.0 International license.

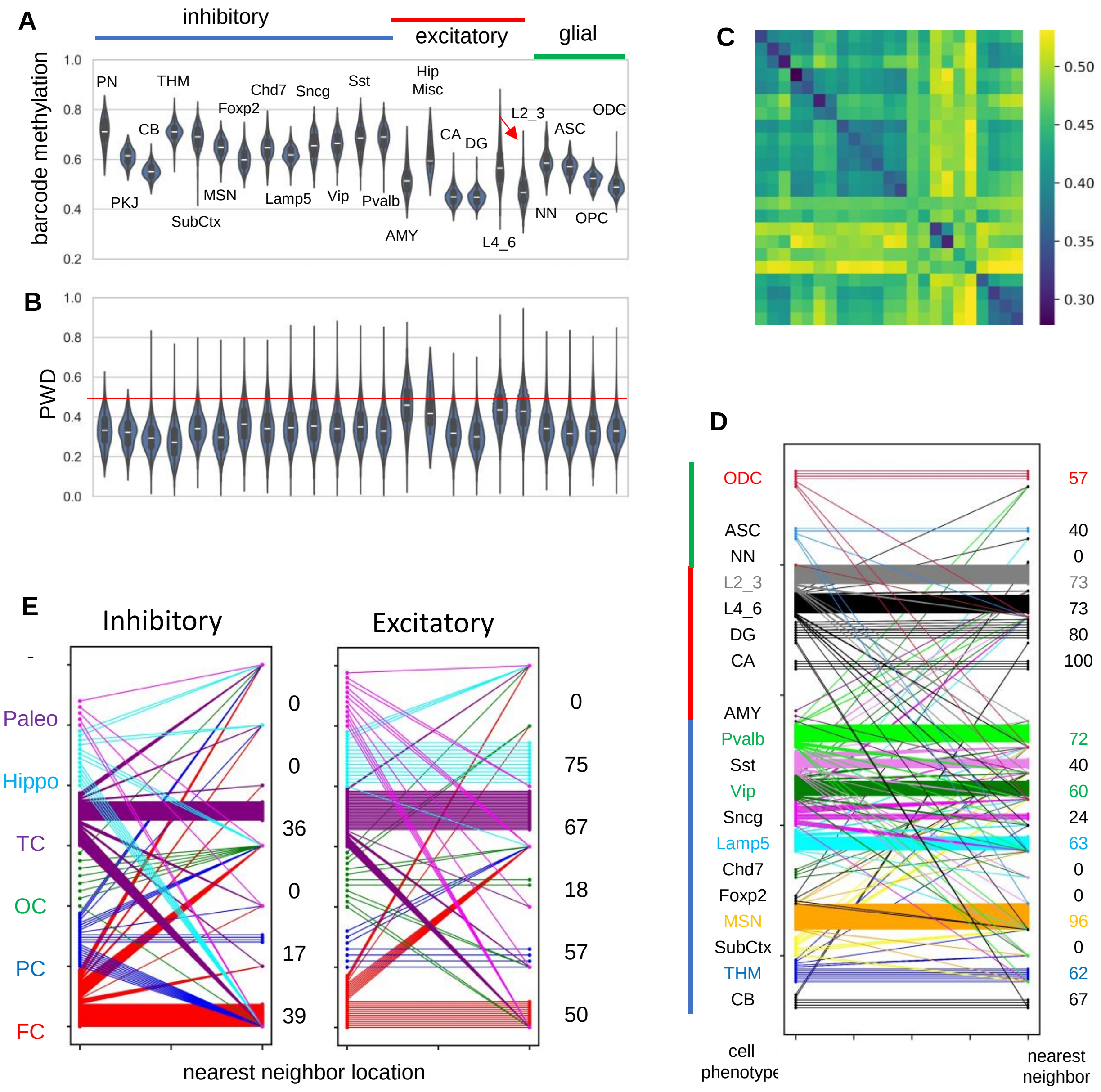

Supplemental Figure 1: H02 data
A) Barcode methylation for different cell types
B) PWDs between cells of the same type
C) PWDs between cell types
D) Lineage cell type fidelity between nearest neighbor pairs (PWD<0.05)
E) Location fidelity between nearest neighbor pairs (PWD<0.05)

bioRxiv preprint doi: https://doi.org/10.1101/2024.07.14.603450; this version posted July 17, 2024. The copyright holder for this preprint (which was not certified by peer review) is the author/funder, who has granted bioRxiv a license to display the preprint in perpetuity. It is made available under aCC-BY 4.0 International license.

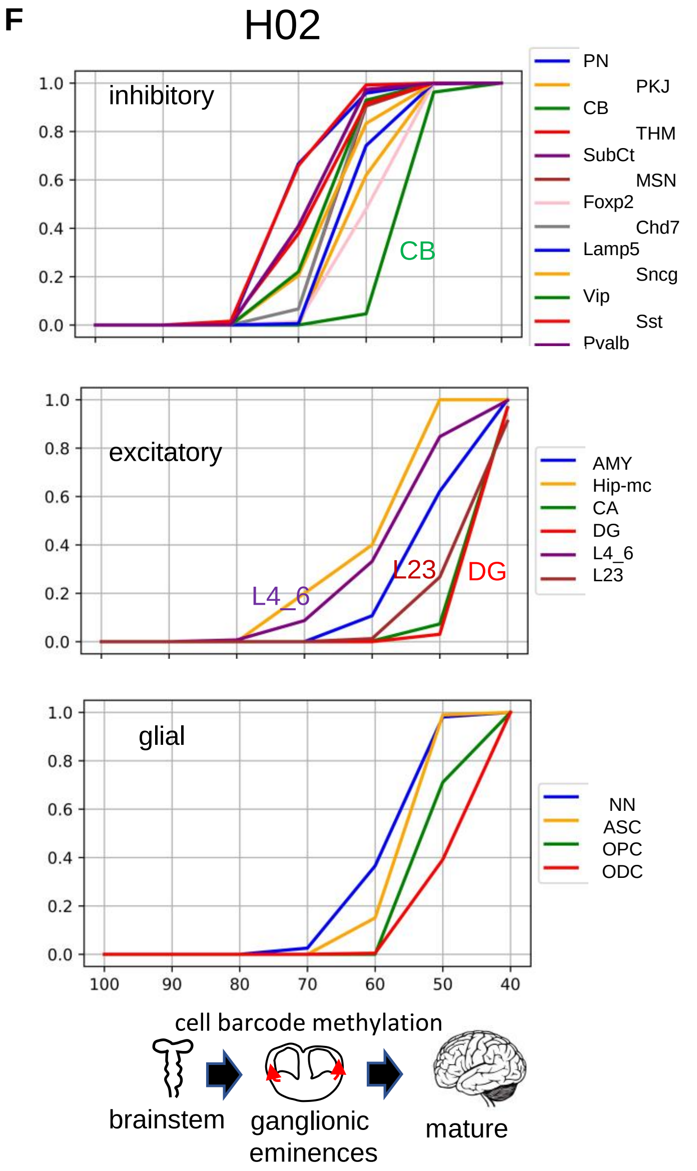

Supplemental Figure 1: H02 data

F) Barcode methylation versus final adult brain content indicates that inhibitory neurons appear first and reach their adult levels before excitatory or glial cells.

bioRxiv preprint doi: https://doi.org/10.1101/2024.07.14.603450; this version posted July 17, 2024. The copyright holder for this preprint (which was not certified by peer review) is the author/funder, who has granted bioRxiv a license to display the preprint in perpetuity. It is made available under aCC-BY 4.0 International license.

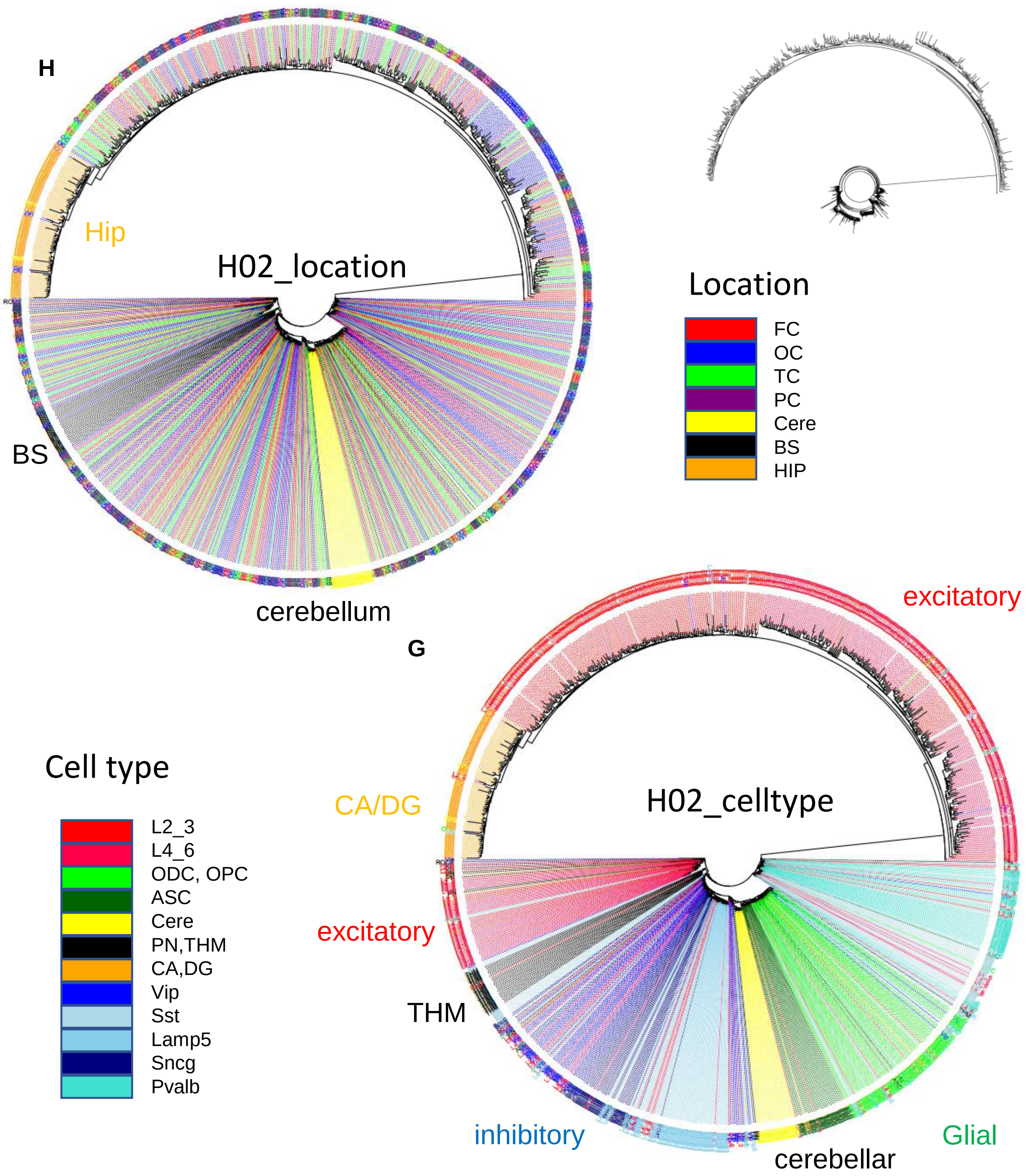

Supplemental Figure 1: H02 data

G) Ancestral tree with 1,001 cells rooted at a fully methylated progenitor shows sequential branching with excitatory, then brain stem, inhibitory and cerebellar neurons, then glial cells, and finally excitatory neurons with hippocampal neurons at the end.

H) Related cells colocalize for brain stem, cerebellar, and hippocampal neurons. Inhibitory neurons are more scattered. Excitatory neurons are also scattered with some localization within the cortex (see I & J for trees with more neurons)

bioRxiv preprint doi: https://doi.org/10.1101/2024.07.14.603450; this version posted July 17, 2024. The copyright holder for this preprint (which was not certified by peer review) is the author/funder, who has granted bioRxiv a license to display the preprint in perpetuity. It is made available under aCC-BY 4.0 International license.

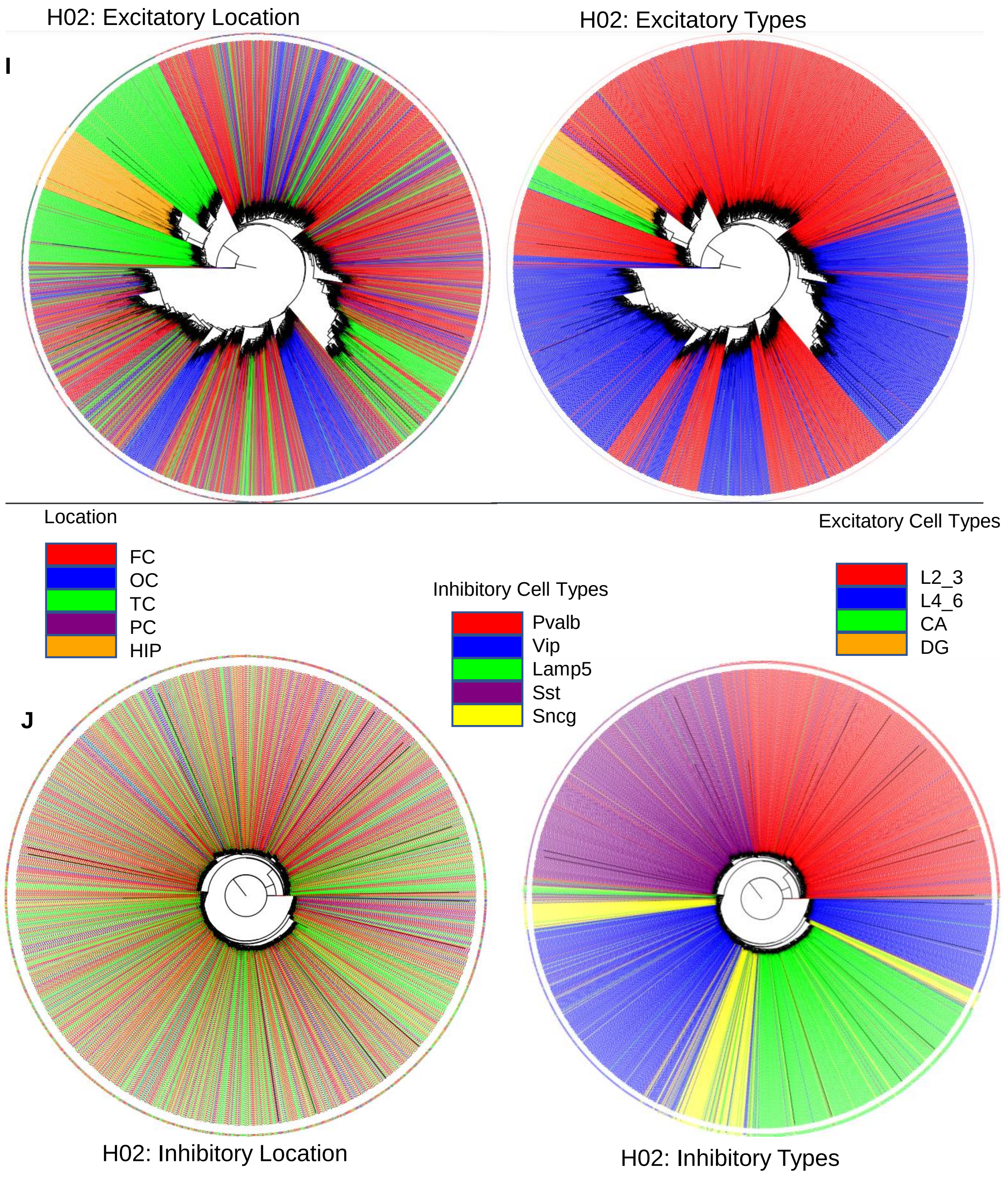

Supplemental Figure 1: H02 data
I) Ancestral tree with more (2,806) excitatory neurons shows more localization between related neurons in the hippocampus, occipital and temporal cortex. Related neurons also tend to have similar phenotypes.
J) Ancestral tree with more (2,788) inhibitory neurons still shows scattering between related neurons. Related neurons tend to have similar phenotypes.

bioRxiv preprint doi: https://doi.org/10.1101/2024.07.14.603450; this version posted July 17, 2024. The copyright holder for this preprint (which was not certified by peer review) is the author/funder, who has granted bioRxiv a license to display the preprint in perpetuity. It is made available under aCC-BY 4.0 International license.

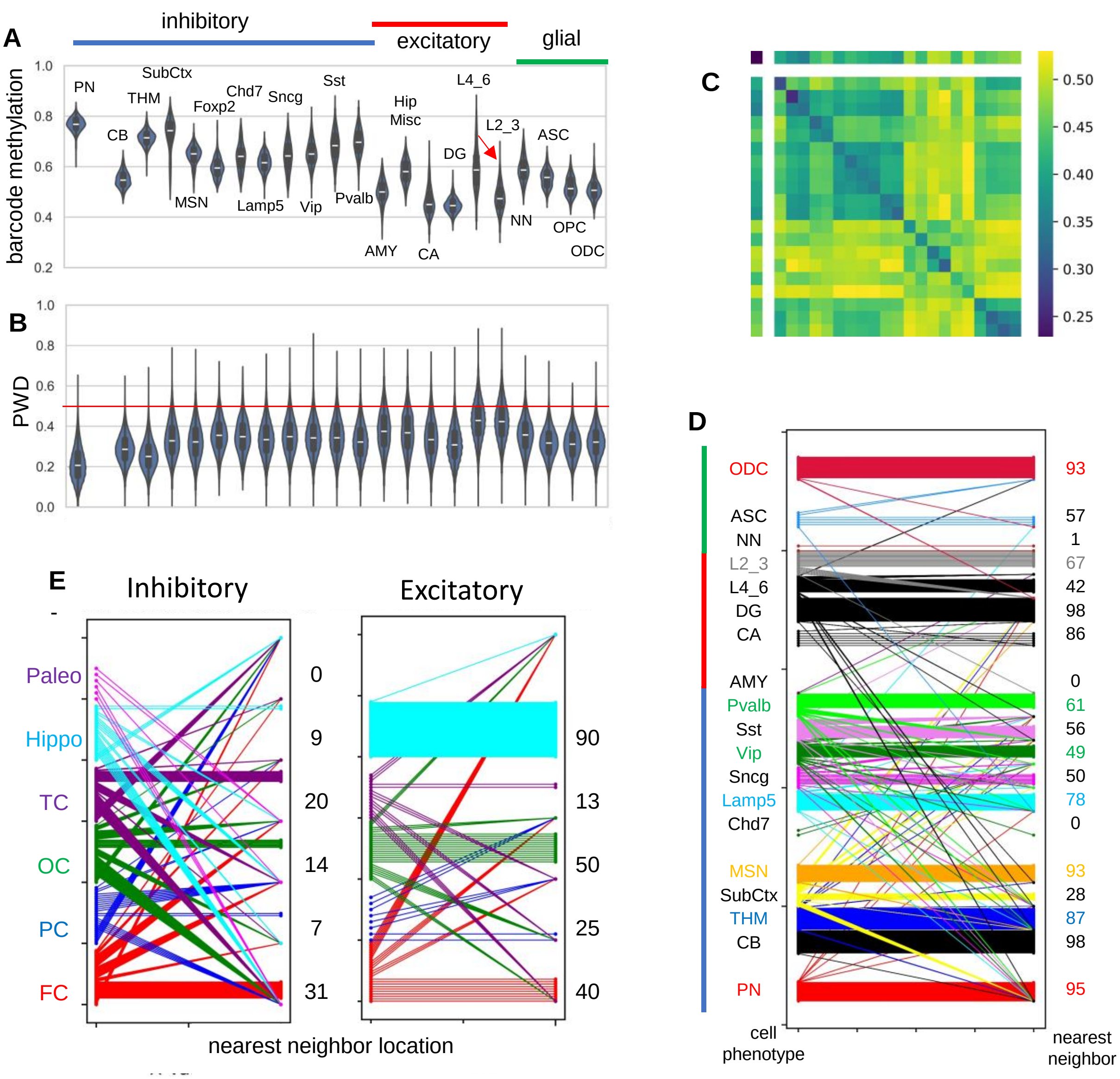

Supplemental Figure 2: H04 data
A) Barcode methylation for different cell types
B) PWDs between cells of the same type
C) PWDs between cell types
D) Lineage cell type fidelity between nearest neighbor pairs (PWD<0.05)
E) Location fidelity between nearest neighbor pairs (PWD<0.05)

bioRxiv preprint doi: https://doi.org/10.1101/2024.07.14.603450; this version posted July 17, 2024. The copyright holder for this preprint (which was not certified by peer review) is the author/funder, who has granted bioRxiv a license to display the preprint in perpetuity. It is made available under aCC-BY 4.0 International license.

F

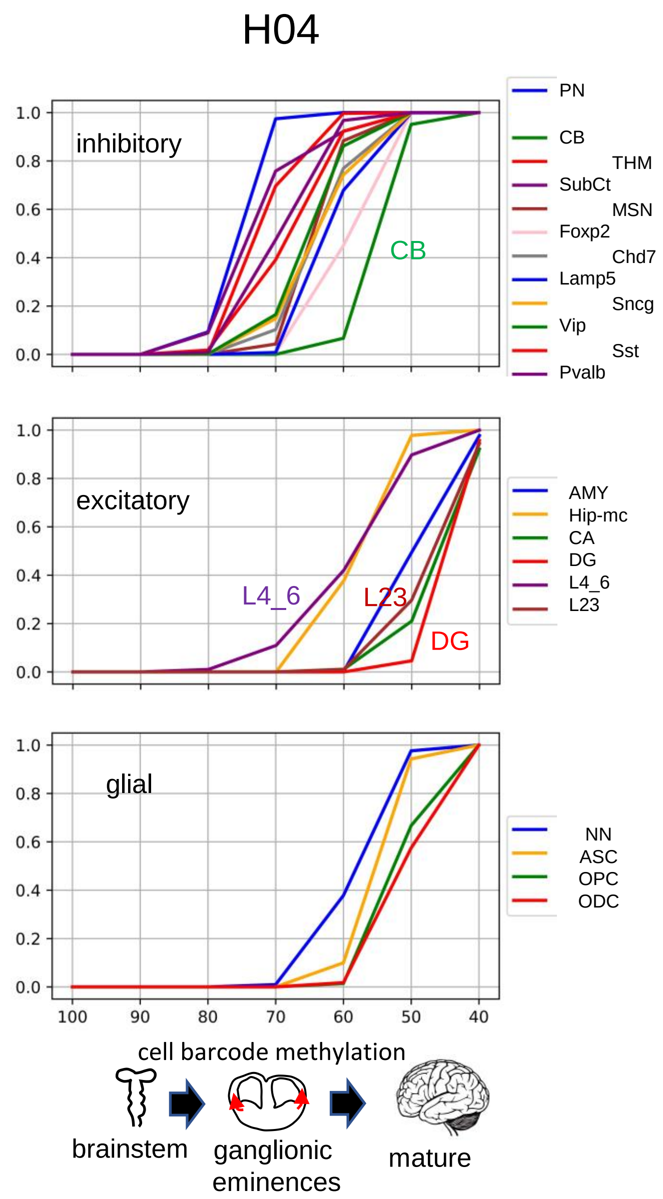

Supplemental Figure 2: H04 data

F) Barcode methylation versus final adult brain content indicates that inhibitory neurons appear first and reach their adult levels before excitatory or glial cells.

bioRxiv preprint doi: https://doi.org/10.1101/2024.07.14.603450; this version posted July 17, 2024. The copyright holder for this preprint (which was not certified by peer review) is the author/funder, who has granted bioRxiv a license to display the preprint in perpetuity. It is made available under aCC-BY 4.0 International license.

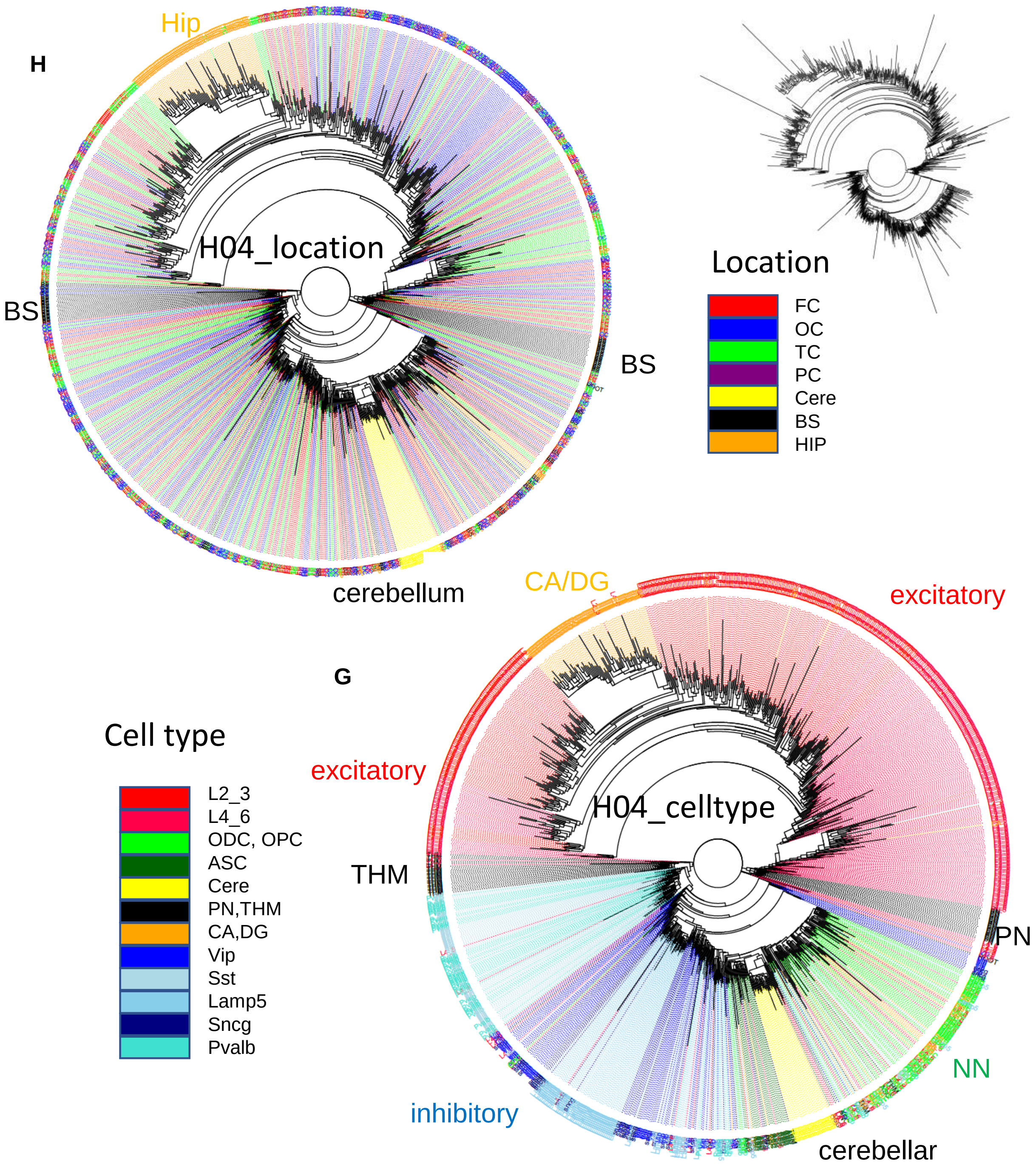

Supplemental Figure 2: H04 data

G) Ancestral tree with 1,033 cells rooted at a fully methylated progenitor shows sequential branching with excitatory, then brain stem, inhibitory and cerebellar neurons, then glial cells, and finally excitatory neurons with hippocampal neurons at the end.

H) Related cells colocalize for brain stem, cerebellar, and hippocampal neurons. Inhibitory neurons are more scattered. Excitatory neurons are also scattered with some localization within the cortex (see I & J for trees with more neurons)

bioRxiv preprint doi: https://doi.org/10.1101/2024.07.14.603450; this version posted July 17, 2024. The copyright holder for this preprint (which was not certified by peer review) is the author/funder, who has granted bioRxiv a license to display the preprint in perpetuity. It is made available under aCC-BY 4.0 International license.

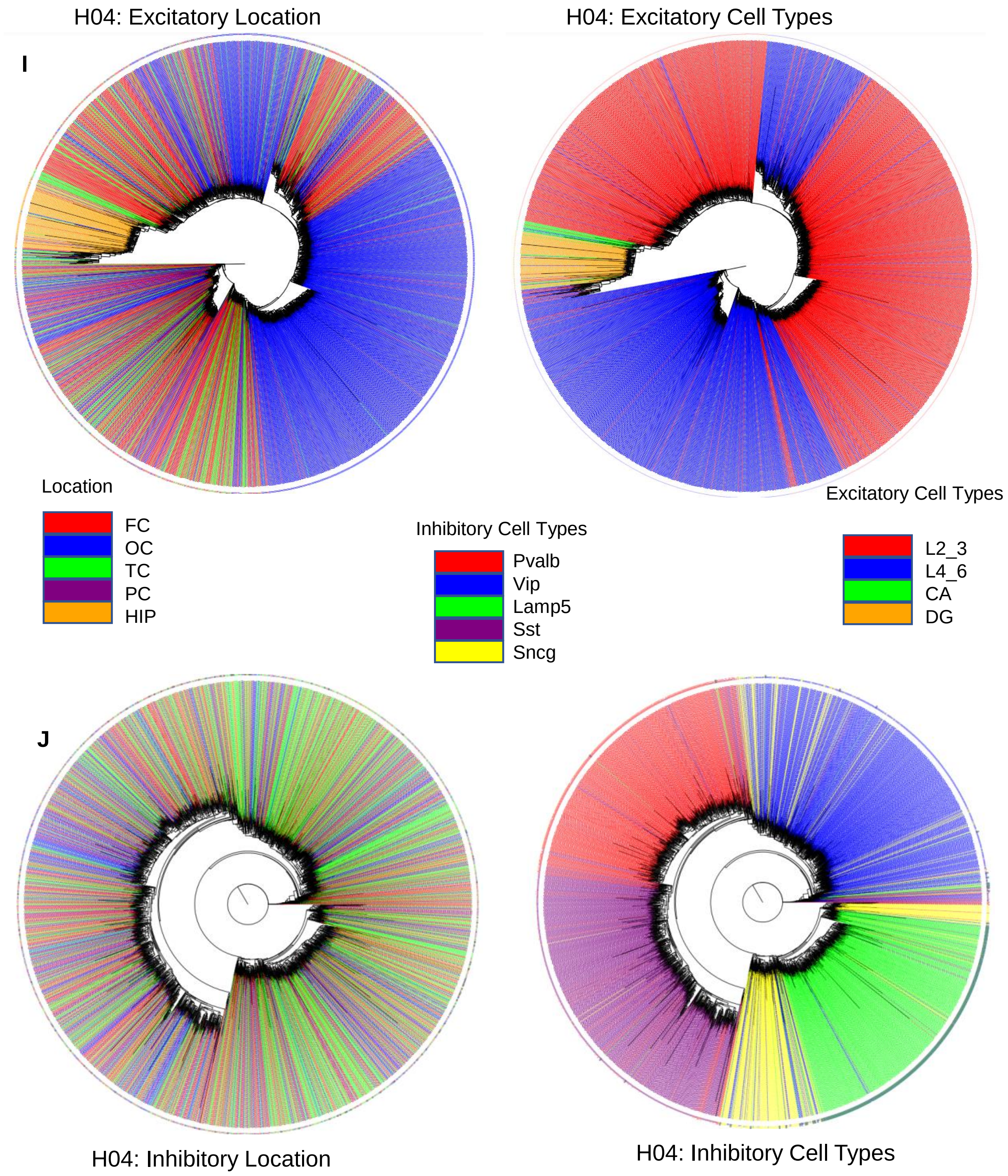

Supplemental Figure 2: H04 data

I) Ancestral tree with more (2,797) excitatory neurons shows more localization between related neurons in the hippocampus, occipital and temporal cortex. Related neurons also tend to have similar phenotypes.

J) Ancestral tree with more (2,752) inhibitory neurons still shows scattering between related neurons. Related neurons tend to have similar phenotypes.

bioRxiv preprint doi: https://doi.org/10.1101/2024.07.14.603450; this version posted July 17, 2024. The copyright holder for this preprint (which was not certified by peer review) is the author/funder, who has granted bioRxiv a license to display the preprint in perpetuity. It is made available under aCC-BY 4.0 International license.

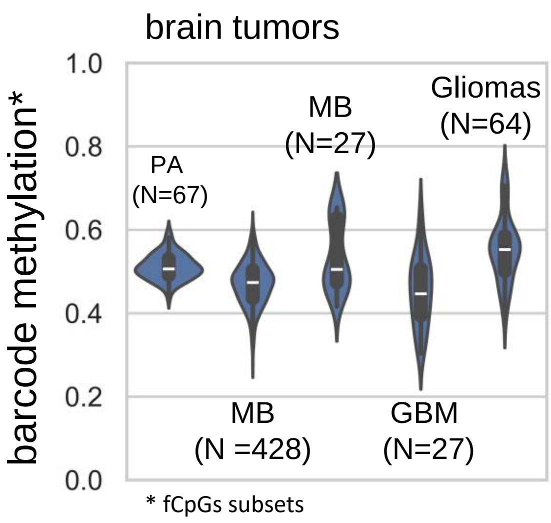

Supplemental Fig 3: Brain tumor fCpG methylation: fCpG sites were matched with brain tumor data from methylation arrays. Average methylation is displayed for each brain tumor sample.
PA= pilocytic astrocytoma, GSE44684, 145 fCpGs covered;
MB=medulloblastoma, GSE193646, 434f CpGs covered;
MB=medulloblastoma, GSE63669, 883 fCpGs covered;
GBM=glioblastoma multiforme, GSE109399, 1,138 fCpGs covered;
Gliomas=various gliomas, GSE248471, 1,138 fCpGs covered.
Includes both male and female tumors.
